# Reply

**DOI:** 10.1002/hep.27393

**Published:** 2015-03-25

**Authors:** Tamer Abdelrahman, Joseph Hughes, Janice Main, John McLauchlan, Mark Thursz, Emma Thomson

**Affiliations:** 1 MRC University of Glasgow Centre for Virus ResearchGlasgow, UK; 2 Department of Medicine, Imperial College NHS TrustLondon, UK

We agree with Martin et al. that, as yet, late recurrence of hepatitis C virus (HCV) following treatment has not been studied in this highly exposed group of patients. To clarify the point made in our study,^1^ the majority of cases labeled as HCV reinfection within the 24-week window posttreatment are likely to represent viral rebound rather than reinfection, even in the presence of a switch in genotype or subtype. In the absence of detailed sequencing data, it indeed seems likely that patients with recurrent HCV infection have a high rate of reinfection,^2^ although there is a need to carry out an appropriately designed study to confirm this, as some studies in other highly exposed cohorts have shown that relapse is associated with recrudescence of similar strains, others have lacked analysis of paired samples, and none have employed a next-generation sequencing approach.^3^,^4^ In the meantime, the proposed adjustment to the reinfection rate following removal of the 7% of patients who relapsed within the 24-week posttreatment would seem entirely appropriate.^5^,^6^ We also agree that retreatment is indicated in patients with recurrent HCV infection. The role of the emergence of resistant variants will be of particular interest as direct-acting antivirals (DAAs) are rolled out, in particular when interferon-free regimens are used, as interferon resistance is likely to be heavily influenced by the host response and is unlikely to occur solely due to mutations within the viral genome.
